# Research on Universal Combinatorial Coding

**DOI:** 10.1155/2014/414613

**Published:** 2014-03-19

**Authors:** Jun Lu, Zhuo Zhang, Juan Mo

**Affiliations:** ^1^College of Computer Science and Technology, Heilongjiang University, Harbin 150080, China; ^2^Key Laboratory of Database and Parallel Computing of Heilongjiang Province, Harbin 150080, China

## Abstract

The conception of universal combinatorial coding is proposed. Relations exist more or less in many coding methods. It means that a kind of universal coding method is objectively existent. It can be a bridge connecting many coding methods. Universal combinatorial coding is lossless and it is based on the combinatorics theory. The combinational and exhaustive property make it closely related with the existing code methods. Universal combinatorial coding does not depend on the probability statistic characteristic of information source, and it has the characteristics across three coding branches. It has analyzed the relationship between the universal combinatorial coding and the variety of coding method and has researched many applications technologies of this coding method. In addition, the efficiency of universal combinatorial coding is analyzed theoretically. The multicharacteristic and multiapplication of universal combinatorial coding are unique in the existing coding methods. Universal combinatorial coding has theoretical research and practical application value.

## 1. Introduction

Coding theory includes three branches: source coding, channel coding, and secrecy coding [1]. The primary mission of source coding is data compression, such as Huffman coding [2], arithmetic coding [3, 4], and dictionary coding [5, 6]. Channel coding can improve the reliability of communication, such as error correcting code. In order to guarantee the information security in the transmission, secrecy coding is used. It can usually be achieved by the data encryption and decryption.

Generally speaking, each existing coding method only belongs to a certain branch of the three coding branches. In fact, relations exist more or less in many coding methods. It means that a kind of universal coding method is objectively existent. This coding method can reflect a variety of encoding features from different points of view and become a bridge connecting many coding methods; thus, the deep development of coding technology can be promoted.

## 2. Theory of Combinatorial Coding

Universal combinatorial coding is a kind of lossless coding method. The concept of “universal” has three meanings: firstly, it means that the encoding method does not depend on the probability statistic characteristic of information source; secondly, it means that this method has multicharacteristic because of its combinatorial and exhaustive properties; thirdly, it refers to the multiapplication.

Assume that a sequence *a*_1_ *a*_2_ ⋯ *a*_*n*_ with *n* elements will be coded, and it has *m* different code elements; the frequency of each element is *n*_*i*_. If the *n* elements are arranged to full permutation according to the benchmark sequence, a new dictionary space which has strict permutation order of sequence can be formed. The problem is how to compute the position of the sequence in the space of the dictionary.

To the *j*th element in the sequence, if it is the same as the *i*th element in the benchmark sequence, it means that in corresponding current position, the existing sequence which is in front of the sequence being coded is already involving *i* − 1 elements; the permutation number of each element *x* in the *i* − 1 elements can be expressed by *S*_*j*,*x*_ when it occupies the *j*th position. (The position of this element in the *i* − 1 element is *x*.) It is shown in (1). Consider

(1) Sj,x=Cn−jw1∗Cn−j−w1w2∗Cn−j−(w1+w2)w3∗⋯∗Cn−j−(w1+w2+⋯+wx−2)wx−1∗Cn−j−(w1+w2+⋯+wx−1)wx−1∗Cn−j−(w1+w2+⋯+wx)wx+1∗⋯∗Cn−j−∑q=1i′−2wqwi′−1∗Cn−j−∑q=1i′−1wqi′.

Each *w* in (1) represents the number of relevant elements. “*i*′” is the number of key elements and the number of each element in these key elements is not 0. Equation (1) involves numerous computations of permutations and combinations; the computational efficiency is too low; therefore, (1) can be optimized to

(2) $$\begin{array}{c} S_{j,x}=\frac{\left(n-j\right)!}{\left(\prod_{q=1}^{x-1}\left(w_{q}!\right)\right)*\left(w_{x}-1\right)!*\left(\prod_{q=x+1}^{i^{\prime }}\left(w_{q}!\right)\right)}. \end{array}$$

To the character *j*th, the total number of sequences involving the *i* − 1 elements in front of the character *j*th is shown in (3). Consider

(3) $$\begin{array}{c} \overset{i-1}{\underset{x=1}{\sum }}S_{j,x}. \end{array}$$

Finally the position of the sequence with *n* element in the dictionary (ordinal) can be computed by

(4) $$\begin{array}{c} o=\overset{n}{\underset{j=1}{\sum }}\;\overset{i-1}{\underset{x=1}{\sum }}S_{j,x}. \end{array}$$

The basic idea of decoding is the presupposition that the measured position is a certain element based on the order of the benchmark sequence. The corresponding permutations value *p*_1_ is calculated based on the assumption. Let *p*_1_ compare with the ordinal number; if the ordinal is equal to *p*_1_ or greater than *p*_1_, then calculate the permutation and combination value (*p*_2_) of the next element according to the benchmark sequence. Judge that whether the ordinal is equal to or greater than *p*_1_ + *p*_2_⋯ until the ordinal is less than *p*_1_ + *p*_2_ + ⋯+*p*_*r*_. At this time, the corresponding element of *p*_*r*_ should be in the current position. Finally, in order to calculate the element of the next position, the new ordinal can be calculated based on

(5) $$\begin{array}{c} o_{\text{new}}=o_{\text{old}}-\left(p_{1}+p_{2}+\cdots +p_{r-1}\right). \end{array}$$

When the value of the ordinal number is 0, the algorithm ends. If it still has remainder elements at this time, then the remaining elements can be filled behind the analyzed sequence according to benchmark sequence.

## 3. Optimized or Parallel Computing

Adopting combinatorial method to calculate the ordinal has higher computational complexity; the calculation process of the ordinal number can be optimized by proportion method.

In the process of the calculation of (3), each permutation and combination value in *i* − 1 elements of the *j*th corresponding group has proportional relation; that is to say, the proportion between each permutation and combination value and the first permutation and combination value *C*_*j*,1_ is equal to the proportion between the number of the corresponding element and the number of the first element. Then (3) can be optimized to

(6) $$\begin{array}{c} \overset{i-1}{\underset{x=1}{\sum }}S_{j,x}=\frac{\left(C_{j,1}*\sum_{k=1}^{i-1}w_{k}\right)}{w_{1}}. \end{array}$$

In fact, there is proportional relation between the permutation and combination value of the first element in the (*j* + 1)th element group and the permutation and combination value of the first element in the *j*th element group. It is shown as follows:

(7) $$\begin{array}{c} \frac{C_{j+1,1}}{C_{j,1}}=\frac{w_{i}}{n^{\prime }}. \end{array}$$

In (7), *w*_*i*_ is the actual frequency of the *j*th character. This character is the *i*th element in the frequency table. Assume that the number of remaining elements is *n*′ (*n*′ = (the number of the sequence element) − *j*). *C*_1,1_ is a particular case, it can be specially processed. Take *C*_0,1_ as the permutation and combination number of all elements for the sequence to be coded; *n*′ is the number of all elements for the sequence to be coded; *w*_*i*_ is the number of the first element in the benchmark sequence.

Of course, it also needs to process some other specific situations, such as for continuous empty elements which is the first element in the benchmark sequence. In the group corresponding the (*j* + empty + 1)th element, the first element permutation value can be expressed as

(8) $$\begin{array}{c} C_{j+\text{empty}+1,1}=C_{j,1}*\frac{t*\left(t-1\right)*\cdots *\left(t-\text{empty}\right)}{n^{\prime }*\left(n^{\prime }-1\right)*\cdots *\left(n^{\prime }-\text{empty}\right)}. \end{array}$$

In (8), *t* represents the number of the first element in the benchmark sequence, when the first *j*th step is processed. It is the current number of the first element in the benchmark sequence. When empty is 0, (8) is (7).

In the process of computation, the first permutation and combination value corresponding to the first element is computed by the whole permutation and combination value Max. *Max*⁡ can be calculated through

(9) $$\begin{array}{c} \mathrm{Max}=\frac{n!}{n_{1}!*n_{2}!*\cdots *n_{m}!}. \end{array}$$

By the proportion method, the computing speed of the ordinal will be greatly improved.

The decoding process of proportion computation is similar.

When value *n* is determined, in order to accelerate the computing speed of Max,  *Max*⁡ can be calculated in advance when all *n*_*i*_ are equal to each other. At this time *Max*⁡ is the largest and is called Whole; then Whole is stored in a file. When it is used, it can be read from the document directly. Then the corresponding adjusted *Max*⁡ is obtained according to the different *n*_*i*_ in actual sequence. Obviously, the Whole can be calculated based on

(10) $$\begin{array}{c} \text{Whole}=\frac{n!}{{\left(\left(n/m\right)!\right)}^{m}}. \end{array}$$

In order to process conveniently, the sequence length *n* is usually integer times of different code element number *m*.

In addition, it can also split the sequence; thus the ordinal number can be computed in parallel and the calculation efficiency will be improved [7].

**Figure 1:**
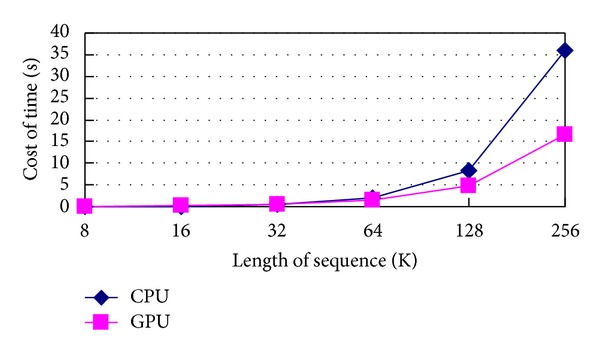
The average time comparison of computing whole ordinal at different lengths between CPU and GPU.

GPU parallel computing is developing rapidly and it can be used in various areas [8, 9]. Thousands of multithread processes can vastly advance the computing speed of the universal combinatorial coding method. But the existing coding methods are difficult to adopt GPU parallel computing technology, such as arithmetic coding or dictionary coding. Because this paper will describe the universal combinatorial coding from the angle of theory, the GPU parallel method need not be given unnecessary details. The average time comparison of computing whole ordinal at different lengths between CPU and GPU is done in Figure 1.

It can be seen in Figure 1 that the slope of CPU serial computing curve is bigger, and the slope of GPU parallel computing curve is smaller. It explains that with the sequence length increasing, CPU serial computation time cost grows fast; GPU parallel computation time cost grows relatively slow. When the length of sequences are 8 K, 16 K, and 32 K, GPU parallel computation time cost is more than time cost of CPU serial computation. For the reason that as the length of sequence is not long enough, the saved time of GPU parallel computation could not make up for the cost time that spends on data transfers between CPU and GPU. When the length of sequence is 64 K, GPU parallel computation time cost starts to be less than time cost of CPU serial computation. It indicates that the saved time of GPU parallel computation is more than the cost time that is spent on data transfer between CPU and GPU. From this time on, the advantage of GPU parallel computing is increasing more and more with the sequence length.

**Figure 2:**
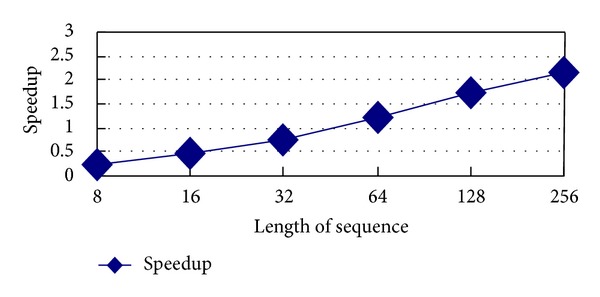
The speedup of GPU parallel computing.

The increasing of speedup is shown in Figure 2.

According to the trend of curve in Figure 2, the longer the sequence length is, the greater the speedup is.

Through the analysis of experiment, obviously, the speed of whole ordinal computing can be improved by GPU parallel technology and running speed increases with the increasing of the sequence length.

## 4. Relation between Universal Combinatorial Coding and Other Coding Methods

Universal combinatorial coding has many features in common with variety of classical encoding methods. (Lots of current encoding methods are derived from these classical encoding methods and are improved according to special application [10–12].) And the preliminary studies have shown that the universal combinatorial coding has characteristics of tree coding, arithmetic coding, and dictionary coding. The following discussion involves the universal combinatorial coding and other coding methods.

### 4.1. Universal Combinatorial Coding Is a Tree Coding

Assume that there is a sequence: *a*_1_ *a*_2_ ⋯ *a*_*n*_, which consists of *m* different elements and contains *n* elements. Thereinto, *m* = 2^*v*^; that is to say, each element occupies *v* bit. Benchmark sequence space contains *m* different elements with full permutation. Obviously, there is *m*! in benchmark sequence. Once a benchmark sequence is confirmed, a *m* tree can be generated, and the position of *a*_1_ *a*_2_ ⋯ *a*_*n*_ in this *m* tree can be confirmed. The other paths from root to leaf node in *m* tree represent the sequences that have the same code element with *a*_1_ *a*_2_ ⋯ *a*_*n*_, but only arrange order is different. Total sequence number is Max; serial number starts from 0, the largest serial number Max − 1 is the biggest ordinal.

**Figure 3:**
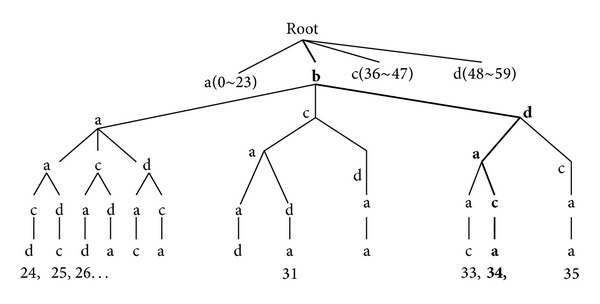
Quad tree which has “bdaca”.

For example, assume the sequence is “bdaca,” different element number is 4. Obviously, *v* is 2. Benchmark sequence number is 4! = 24. It means that there are 24 trees which are all quad tree with strict order. Assume that benchmark sequence is “abcd,” then each code element can be represented as a: 00, b: 01, c: 10, and d: 11. Thus the sequence can be expressed as 0110001100 and occupy 10 bits. If this sequence uses tree form, the tree can be obtained as Figure 3.

The quad tree in Figure 3 consists of sequences that each sequence contains two “a,” one “b,” one “c,” and one “d.” The sum of sequences is *C*_5_^2^∗*C*_3_^1^∗*C*_2_^1^∗*C*_1_^1^ = 60. These sequences strictly arrange in accordance with order. Serial number is from 0 to the biggest ordinal number 59. The ordinal number corresponding to the sequence “bdaca” is 34; this ordinal number stands for the number of the sequences in front of “bdaca” and has the same code element but has different rank place. So the “bdaca” position in the quad tree can also reflect the property of combination. Decoding process is vice versa.

Thus, combinatorial coding can be expressed as a tree structure. It calculates the coding sequence position in *m*-tree structure based on the principle of combinatorial theory.

### 4.2. Universal Combinatorial Coding Is Dictionary Coding

It can arrange the path of *m*-tree according to the order from left to right strictly, a dictionary can be obtained. It can be scripted as follows: assume that there is a sequence: *a*_1_ *a*_2_ ⋯ *a*_*n*_, which consists of *m* different elements. Thereinto, *m* = 2^*v*^ and each code element occupies *v* bit. The presence times of code elements are *n*_1_, *n*_2_ ⋯ *n*_*m*_. Obviously, *n*_1_ + *n*_2_ ⋯ *n*_*m*_ = *n*. And the benchmark sequence is made up of *m* different code elements, the number of the benchmark sequence is *m*!. Once the benchmark sequence is confirmed, a dictionary space can be determined, and the position (ordinal number) of *a*_1_ *a*_2_ ⋯ *a*_*n*_ in the dictionary space is also determined. The dictionary space stores all the sequences that have the same code element and different permutation order with *a*_1_ *a*_2_ ⋯ *a*_*n*_. The number of sequences can be calculated according to (9).

The ordinal of the coding sequence can also be calculated according to the related equations in the sections ahead. The whole dictionary space and the position of the coding sequence in the dictionary can be shown in Table 1.

Each sequence in the dictionary has strict order with the constraints of the benchmark sequence. Of course, the combination dictionary is* nonexistent*. It is hidden in the frequency table of the sequence. It means that it does not require actual occupation of space and time. The position (ordinal number) of the coding sequence in the dictionary can be calculated, and the size of the ordinal number is less than the size of sequence space. Taking advantage of this characteristic, universal combinatorial coding can be used for data compression. The dictionary space of the universal combinatorial coding is fixed and is objectively existent. This dictionary contains all the sequences that have the same code number with the coded sequence.

### 4.3. Universal Combinatorial Coding Is Arithmetic Coding

Universal combinatorial coding uses an absolute position value to express sequence in the dictionary space and this value is an integer; so it can be seen as an arithmetic coding. In fact, the traditional arithmetic coding expresses a sequence as a digit which is a real data between 0 and 1. It can be understood as a relative position value.

Another method which is closer to the universal combinatorial coding is range encoding method. The range encoding method can also be seen essentially as arithmetic coding. But the range encoding method must have a large enough positive integer. In fact, the so-called sufficient large positive integer plays the role of the largest ordinal number, but it is not precise enough and it is larger than the actual needs of the largest ordinal number. In other words, ordinal number of the universal combinatorial coding is actually the smallest and most accurate “positive integer which is large enough.”

The most important thing is that both range coding and 0-1 arithmetic coding are based on probability, and we always assume that the probability is constant. It will inevitably lead to error. Even worse, in many cases, the probability of sequence elements cannot be predicted. Of course, adaptive arithmetic coding may not depend on the probability, but its compression ratio is reduced accordingly.

On the contrary, the universal combinatorial coding is not based on probability but based on the frequency. It can adjust the frequency timely in the computation process according to the actual situation. So the accuracy of the ordinal number can be ensured. Of course, the drawback is that the frequency value of each element must be recorded.

**Figure 4:**
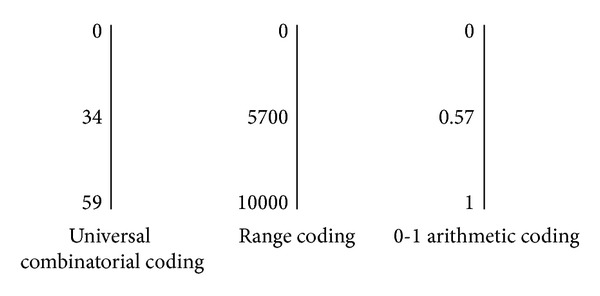
The result of each arithmetic coding.

Figure 4 is a schematic diagram of the calculated results of the instance “bdaca” according to combinatorial coding, region coding (0 to 10000), and 0-1 arithmetic coding.

In summary, universal combinatorial coding uses an integer value to represent the sequence more accurately, while the traditional arithmetic coding methods adopt the relative position or similar position to express the sequence. In fact, if it adopts a proportion value to represent the relative position of the sequence in the entire dictionary space in the universal combinatorial coding, then the proportion is similar to the result of 0-1 arithmetic coding: 34/59 ≈ 0.576.

The combination feature of universal combinatorial coding makes this method have more or less relation with many other coding methods. It means that this method has multicode connectivity. This means that universal combinatorial coding has the potential to become a tool to measure the characteristics of a variety of coding methods.

## 5. Multiapplication of Universal Combinatorial Coding

Universal combinatorial coding also has more special properties besides tree structure coding, arithmetic coding, and dictionary coding. These properties make universal combinatorial coding applicable in many ways.

### 5.1. The Estimation of the Size of the Ordinal

Making use of the relation between the ordinal and the sequence characters frequencies, the size of the ordinal can be estimated preliminarily.

Current research shows that the more frequency differences among the characters in sequence are, the less the dictionary including the sequence is, at the same time, the less the maximum ordinal is and the less the average ordinal is. When the frequency differences among the characters are less than 1, global maximum ordinal can be obtained. The global maximum ordinal is only related to the sequence length. The sizes of the sequence and all kinds of ordinal satisfy the following inequality:

(11) $$\begin{array}{c} \text{le}{\text{n}}_{\text{sequence}}\geq \text{le}{\text{n}}_{\text{Whole}}\geq \text{le}{\text{n}}_{\mathrm{Max}}\geq \text{le}{\text{n}}_{\text{ordinal}}. \end{array}$$

**Figure 5:**
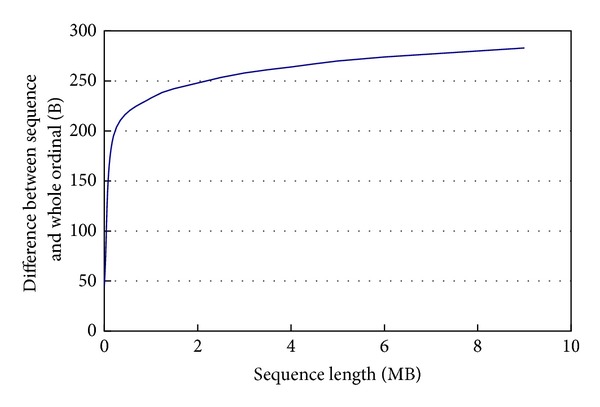
Difference of sequence length and whole ordinal length.

The length of len_sequence_ − len_Whole_ is growing with the growing of the sequence length *n*, but the increasing range is smaller and smaller. It can be seen in Figure 5.

The global maximum ordinal is used for preliminarily estimates or theoretical analysis of the ordinal. In order to calculate the maximum ordinal, it can be precalculated and put in the file. And the maximum ordinal can be used for calculating ordinal of the sequence to be coded.

### 5.2. Combinatorial Compression Property

The property that ordinal length must be less than sequence length can be used for combinatorial compression.

**Figure 6:**
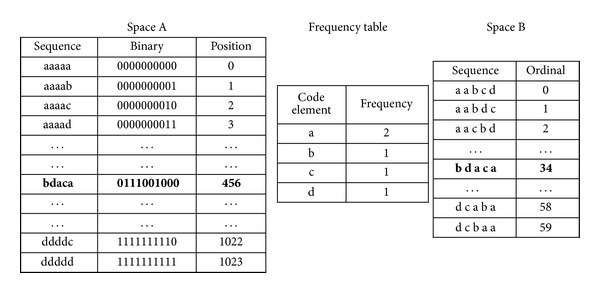
Space map under the benchmark sequence “abcd.”

Universal combinatorial coding mainly utilizes ordinal to reduce frequency redundancy. It codes the whole sequence. The space A can be determined by the length of coding sequence. The space A can be transformed to a smaller space B through the restriction of the sequence frequency table. Space B is made up of sequence which has the same frequency coding table with coding sequence. The position of coding sequence in space B is smaller than the position in space A. For example, to the sequence “bcada,” the number of different elements is 4, each code occupies 2 bits (a: 00; b: 01; c: 10; d: 11). Assume that the benchmark sequence is “abcd,” then the position in space A is 0110001100 (396 in decimal). After being constrained by the frequency table (a: 2; b: 1; c: 1; d: 1), the dictionary space B is formed, and the position of the sequence in space B is 34. It can be shown in Figure 6.

It can be seen from Figure 6 that the space B through the restriction of the sequence frequency table is smaller than the original sequence space A. At the same time, the average position number (ordinal number) of coding sequence in space B also becomes smaller.

### 5.3. Combinatorial Encryption

Make use of the characteristic that ordinal number is not unique, universal combinatorial coding can be used for confidential treatment of data.

At this time the cipher key space is the combination of different code elements in the benchmark sequence (assume that each code element length is *m*). The cipher key number in cipher key space is (2^*m*^)!. When *m* arrives at a certain length, for example, *m* is 6, it can achieve confidential requirement. At this time the cipher key space is (2^6^)!≈1.27∗10^89^, and cipher key space is larger than the current existing encryption algorithm. (They usually use bit as encryption unit [13–15]. When a key length is 256 bits, the cipher key space is 2^256^ ≈ 1.16∗10^77^.) It can achieve the confidential requirement.

**Figure 7:**
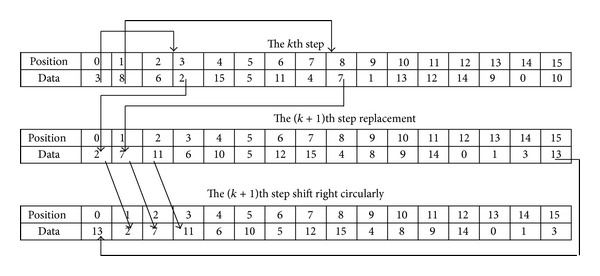
Data and position replacement method.

The arrangement of the key in combination coding adopts the method that data and position are replaced. It is to say, the (*k* + 1)th rounds key can be deduced from the *k*th rounds key; the method is to find out the data *j* in the *i*th location (counting from 0) of the *k*th rounds key first; then take the data *j* as the data of the *i*th location in the (*k* + 1)th rounds key. In order to prevent the appearance of dead circulation of location and data, the result of the (*k* + 1)th rounds key can be shifted right circularly. At last, the final (*k* + 1)th rounds key is formed. It can be shown in Figure 7 (to describe conveniently, take *m* = 4).

**Figure 8:**
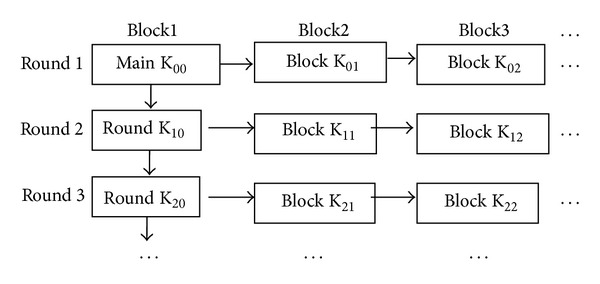
Relation of all kinds of keys.

Data and position replacement method makes each key different, not only round key, but also group key in each round. The creating method of group key is similar to the creating method of round key, only perform right shift to the previous key in turn, and then replace it between data and position.  Figure 8 shows the relationship among the main key, the round key, and the group key.

The key generation method of combinatorial encryption makes the main key, round key, and the group key have the same large space; so the difficulty of decryption is increased further. In addition, combinatorial encryption method adopts code element as the information processing unit instead of bit, so the group length of sequence can be longer, and it is more suitable to parallel computing than the existing encryption algorithm through the multi-core and multi-GPU.

**Figure 9:**
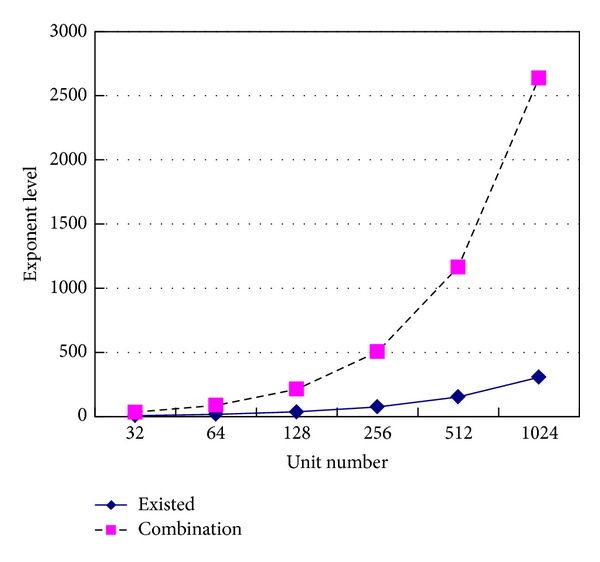
Key space of different encryption methods.

The key space can be compared between the combinatorial encryption method and the existing encryption method (they usually use bit as encryption unit) in Figure 9. In order to express conveniently, there is only exponent level of key space expressed in *y*-axis in Figure 9.

It can be seen form Figure 9 that the key space of combinatorial encryption method expands rapidly along with the increase of code element bits. So security is increased.

### 5.4. The Other Application of Combinatorial Coding

Universal combinatorial coding can also do the simple data detection by using ordinal as error-detecting code. It can be used in data validation of communication transmission or storage information. Universal combinatorial coding can also be used for processing information abstract by incorporating the combinatorial verifying method and secret key. The information abstract is only used for checking whether the data has been distorted, and the data is not necessary to revert. According to the key sequence, multiple combination calculation can be done to the sequence. That is to say that another similar ordinal calculation can be done for the ordinal received every time until the length of ordinal is fit for user requirement. At this moment, the last ordinal is used as the information abstracts. Of course, the times of ordinal calculation must be recorded. For the method of information abstracts computing through universal combinatorial coding; the ratio of collision is very low in theory.

## 6. The Assessment of Universal Combinatorial Coding Efficiency

Universal combinatorial coding is independent of the probability statistical properties of the information source, so Shannon Entropy cannot be used to assess the coding quality for universal combinatorial coding. But most of the evaluation methods still use probability recently; so probability is still adopted to assess universal combinatorial coding efficiency by approximate calculation in this paper. To contrast with Shannon Theory, suppose that the source is a smooth with no memory sequence of *q* elements and the sequence length is *n*. Further, the probability distribution of the source symbols is *p*_*i*_  (*i* = 1,…, *q*). Information source is divided into *C*(*n*) segments to process. When the length of the sequence *n* and the segment length *K* are very large, average code length can be calculated by the following methods:

(12) $$\begin{array}{c} n=KC\left(n\right). \end{array}$$

For each segment whose length is *K*, the number of ordinal can be obtained by universal combinatorial coding as the following formula:

(13) $$\begin{array}{c} N_{k}=\frac{K!}{\left(\prod \left(p_{i}K\right)!\right)}. \end{array}$$

So the length of the binary code occupied by each ordinal is

(14) $$\begin{array}{c} l=\left\lceil \log N_{k}\right\rceil =\left\lceil \log\left(\frac{K!}{\left(\prod \left(p_{i}K\right)!\right)}\right)\right\rceil . \end{array}$$

Although the frequency of each character also occupies space, it is very small compared to the space of ordinal. So the total code length of the sequence with *n* element is

(15) $$\begin{array}{c} C\left(n\right)\left\lceil \log\left(\frac{K!}{\left(\prod \left(p_{i}K\right)!\right)}\right)\right\rceil . \end{array}$$

Finally, the average code length of each source symbol is

(16) $$\begin{array}{c} \overset{-}{L}=\frac{\left(C\left(n\right)\left\lceil \log\left(K!/\left(\prod \left(p_{i}K\right)!\right)\right)\right\rceil \right)}{n}. \end{array}$$

Equation (16) can be rewritten to the inequality of $\bar{L}$ as (17)

(17) $$\begin{array}{c} \overset{-}{L}\geq \frac{\left(C\left(n\right)\left[\log\left(K!/\left(\prod \left(p_{i}K\right)!\right)\right)\right]\right)}{n},\\ \overset{-}{L}≺\frac{\left(C\left(n\right)\left[\log\left(K!/\left(\prod \left(p_{i}K\right)!\right)\right)+1\right]\right)}{n}. \end{array}$$

Put (12) into inequality (17),

(18) $$\begin{array}{c} \frac{\log\left(K!/\left(\prod \left(p_{i}K\right)!\right)\right)}{K}\leq \overset{-}{L},\\ \overset{-}{L}≺\frac{\left(\log\left(K!/\left(\prod \left(p_{i}K\right)!\right)\right)+1\right)}{K}. \end{array}$$

After log-calculation, the results of *N*_*k*_ = *K*!/(∏(*p*_*i*_ *K*)!) is similar to the result of *K*!/(∏(*p*_*i*_ *K*)!); so the following computing can be done:

(19) log⁡(K!(∏(piK)!))≈log⁡(KK(∏(piK)piK))=log⁡KK−log⁡∏(piK)piK=Klog⁡K−(p1Klog⁡(p1K)+p2Klog⁡(p2K)  +⋯+pqKlog⁡(pqK))=Klog⁡K−(K∑pilog⁡pi      +(p1+p2+⋯+pq)      ×Klog⁡K)=−K∑pilog⁡pi=KH(S).

Put (19) into inequality (18),

(20) $$\begin{array}{c} H\left(S\right)<\bar{L}<H\left(S\right)+\frac{1}{K}. \end{array}$$

When *K* is large enough, the average code length can be scripted by the following formula:

(21) $$\begin{array}{c} \bar{L}\approx H\left(S\right). \end{array}$$

It shows that the average code length in the universal combination coding is progressively close to the source entropy limit when *K* is large enough.

But it should be noticed that there is an approximate calculation in the above estimation process. Consider

(22) $$\begin{array}{c} \log\left(\frac{K!}{\left(\prod \left(p_{i}K\right)!\right)}\right)\approx \log\left(\frac{K^{K}}{\left(\prod {\left(p_{i}K\right)}^{p_{i}K}\right)}\right). \end{array}$$

So the calculation result is magnified. It means that the actual coding efficiency is better than the approximate calculation result.

## 7. Conclusions

The concept of universal combinatorial coding is advanced and the related properties are analyzed in this paper. Universal combinatorial coding can stride over the three branches of coding theory. It has the characteristics of multiple coding methods and various application prospects, for example, combinatorial compression, encrypt or decrypt, and error detection. Theoretical researches reveal that the average code length in universal combinatorial coding is close to the source entropy limit when the value of *K* is large enough. But the actual coding efficiency should be better than the approximate calculation results.

## Figures and Tables

**Table 1:** Dictionary space and the position of the coded sequence.

Ordinal	Sequence
0	a a b c d
1	a a b d c
2	a a c b d
⋮	⋮
33	b d a a c
**34**	**b d a c a**
35	b d c a a
⋮	⋮
57	d c a a b
58	d c a b a
59	d c b a a

Bold refers to the position of the example “bdaca” in the dictionary is 34. The size of the dictionary is 60(0~59). It is similar to the example “bdaca” in Figure 3.
